# Hospitalization for acute heart failure: the in-hospital care pathway predicts one-year readmission

**DOI:** 10.1038/s41598-020-66788-y

**Published:** 2020-06-30

**Authors:** Claire Duflos, Pénélope Troude, David Strainchamps, Christophe Ségouin, Damien Logeart, Grégoire Mercier

**Affiliations:** 1 Department of Medical Information, CHU, University of Montpellier, Montpellier, France; 2 PhyMedExp, U1046, INSERM, Montpellier, France; 3 Public Health Department, Universitary Hospital Saint-Louis – Lariboisière – Fernand-Widal, AP-HP, Paris, France; 4 Cardiology Department, Universitary Hospital Saint-Louis – Lariboisière – Fernand-Widal, AP-HP, Paris, France; 5 CEPEL, University of Montpellier, Montpellier, France

**Keywords:** Cardiology, Health services

## Abstract

In patients with heart failure, some organizational and modifiable factors could be prognostic factors. We aimed to assess the association between the in-hospital care pathways during hospitalization for acute heart failure and the risk of readmission. This retrospective study included all elderly patients who were hospitalized for acute heart failure at the Universitary Hospital Lariboisière (Paris) during 2013. We collected the wards attended, length of stay, admission and discharge types, diagnostic procedures, and heart failure discharge treatment. The clinical factors were the specific medical conditions, left ventricular ejection fraction, type of heart failure syndrome, sex, smoking status, and age. Consistent groups of in-hospital care pathways were built using an ascending hierarchical clustering method based on a primary components analysis. The association between the groups and the risk of readmission at 1 month and 1 year (for heart failure or for any cause) were measured via a count data model that was adjusted for clinical factors. This study included 223 patients. Associations between the in-hospital care pathway and the 1 year-readmission status were studied in 207 patients. Five consistent groups were defined: 3 described expected in-hospital care pathways in intensive care units, cardiology and gerontology wards, 1 described deceased patients, and 1 described chaotic pathways. The chaotic pathway strongly increased the risk (p = 0.0054) of 1 year readmission for acute heart failure. The chaotic in-hospital care pathway, occurring in specialized wards, was associated with the risk of readmission. This could promote specific quality improvement actions in these wards. Follow-up research projects should aim to describe the processes causing the generation of chaotic pathways and their consequences.

## Introduction

The prevalence of heart failure (HF) is estimated at 1–2% in Western countries, and its incidence is estimated at 5 to 10 new cases per 1000 person-years. The prevalence of HF is increasing, and this phenomenon is explained in part by the aging of the population. HF is responsible for 1–5% of hospitalizations in Western countries and 30% of avoidable hospitalizations^1^, and HF remains the leading cause of hospitalization for people over 65 years of age^2^. In patients with HF, the incidence of hospitalizations for HF is 12–45% at one year^3,4^, and the incidence of all-cause hospitalizations is 63%^4^.

Clinical and sociodemographic features are known risk factors for hospitalization: age, sex, comorbidities, number of recent ED visits or hospitalizations, loneliness, and social deprivation measured by income, education, or ZIP code of residence^5–9^. Some characteristics of the health care system are also risk factors, especially disparities in access to care^10^: the rate of hospitalizations increases with number of and access to general practitioner (GP), number of acute care bed and number of nurses^1,11^, and self-reported low access to primary care and satisfaction with primary care^12,13^. Practice styles also were associated with the risk of hospitalization including the continuity of care, length of stay, ward’s specialty, and discharge hour^6,8,14–16^. These features can probably explain less assessed risk factors such as adherence to medication^17^ and clinical features at discharge^18^. These results led the health care providers to propose organizational interventions in the community care setting, especially after hospitalizations^19^. The impact of the organization on the in-hospital care has been assessed only in a dispersed manner. Until now, the literature has not dwelled on a refined description of the practice styles in hospitals. Are there differences in the minimal set of investigations and medications prescribed for patients with HF in different wards? Are patients often transferred between wards leading to more challenges in the coordination of care?

The main objective of this study was to characterize the main types of in-hospital pathways for the management of patients in heart failure. The secondary objective was to determine the prognosis value of this typology on the risk of rehospitalization for heart failure at 30 days or 1 year and for all-cause rehospitalization at 30 days or 1 year.

## Methods

### Settings and study design

This retrospective study was carried out in the University Hospital Lariboisière in Paris, France. This hospital houses one of the largest emergency departments (ED) in Paris (60,000 referrals per year), a short stay unit (SSU), and a cardiology ward with an intensive cardiac care unit (ICCU). ICCU typically manages patients with acute heart failure or myocardial infarction during their first days of hospitalization. Patients with cardiogenic shock and multiorgan deficiencies are typically managed in the intensive care unit (ICU). This structure provides basic care for patients within a narrow area (HF) and specialized care for patients in a broader area (HF accompanied by multiorgan deficiencies). In this retrospective, single-center study, a senior physician collected clinical data from the medical charts and the medico-administrative charts of patients of Lariboisière in 2013, and an experienced pharmacy resident collected data regarding their treatment. In accordance with French law, the authors informed the patients of the study and of their right to decline, obtained the ethical authorization from the Institutional Review Board of University Hospital of Montpellier (authorization number 2019_IRB-MPT_07–16) and obtained the authorization to set the database up from the National Committee for Informatics and Liberty (authorization number 1777475 v 0).

### Patients

All admitted patients classified in the diagnostic-related group (DRG) “heart failure or cardiogenic shock” (04M09) present in the claim data of our hospital in 2013 were assessed for inclusion by a senior physician. The causes for admission were confirmed with clinical data, and only the first hospitalization of the year for each patient was included. We did not consider a wash-out period because the concepts studied are relevant for any admission for acute heart failure, were they admission or readmission. We included only patients older than 65 years old because younger patients are rare in this DRG, and age introduces much heterogeneity regarding readmission risk^9^. Lowering heterogeneity of confounding factors allows to produce more accurate estimates of the impact of in-hospital pathways Therefore, excluding young patients was expected to improve the accuracy of our analysis. Stays shorter than 2 days were excluded because these patients were frequently transferred to other hospitals, preventing us from studying their complete in-hospital pathway.

### Variables

Regarding patient demographics and global health status, we collected patients’ age, sex, body mass index, current tobacco use, and data related to the following set of comorbidities: high blood pressure (HBP), chronic obstructive pulmonary disease (COPD), diabetes, renal failure, and atrial fibrillation. We also collected their zip codes and identified the patients living in the Lariboisière’s catchment area. All these factors are known predictors of HF patients’ pronostic^5–9^.

Regarding cardiac decompensation, we collected the following data: syndrome (acute pulmonary edema, congestive heart failure, cardiogenic shock, or right heart failure), left ventricular ejection fraction (LVEF), and blood level of B-type natriuretic peptide (BNP). Indeed, these features lead to different diagnostic and therapeutic management^20^, and are therefore needed to analyse in-hospital care pathways. Regarding the in-hospital management of the patients, we collected the key elements of acute HF management^20^: 1/ the realization of diagnostic tests during hospitalisation: echocardiography, chest X-ray, BNP measurement, creatininemia measurement, and 2/ the prescription of HF treatments at discharge: loop diuretics, angiotensin-converting enzyme inhibitors or angiotensin receptor blockers (ACE-I/ARB), and beta-blockers (BB).

Regarding the in-hospital care pathway, we collected the admission type (from home, from the ER, or transferred from another hospital), the discharge type (to home, to another acute care hospital, to a rehabilitation center, or to a long-term care hospital), the number of wards where the patients stayed during his/her hospitalization, and their passage to the SSU, cardiology ward, ICCU, ICU, and geriatric ward. We also collected the length of stay (LOS). Because LOS depends mainly on the clinical status of the patient, we defined a long stay as a LOS in the upper quartile, stratified on the severity of the patient, as graded by the national diagnostic-related groups algorithm. In the four increasing severity groups, the upper quartile of LOS was respectively of 8, 10, 16 and 31 days. All these variables are alleged organizational factors which may ease or impair the delivery of adequate management. Some of them were already assessed^6,8,14–16^.

Regarding the outcomes, we collected readmissions in all hospitals for heart failure or any cause within 30 days or 1 year after discharge.

### Statistical analysis

We designated homogenous groups of patients using a hierarchical ascendant classification (HAC) based on a multiple correspondence analysis (MCA) [22]. This cluster analysis discerns patterns and creates groups that have similar characteristics across clustering variables. Such methods have already been successfully used to analyze claim data [23] and to classify physician practice styles [14] (see Box 1 and Appendix for details). To address our principal aim, we used the in-hospital care pathway and management variables as our clustering variables. There was no missing data for these variables. Then, all descriptive statistics for all variables were generated according to groups in order to show the main types of in-hospital care pathways existing in this setting and to determine which types of patients are associated with each in-hospital pathway.

Because our outcomes were in the form of count data, we used negative binomial regression models to test the effect of the in-hospital care pathway. For potential confounders, all clinical variables were included in the models. We could not include BNP, creatinine, or BMI due to missing data. The smoking status of a patient was classified as never smoker, former smoker, current smoker, or unknown. Then, all variables with a p-value lower than 0.1 were selected. The details are provided in the Supplementary data.

The significance threshold was set at 0.05 for all analyzes. No sex-based or race/ethnicity-based differences were presented.

Box 1 Methodological Box Clustering methods (e.g. Hierarchical Ascendant Classification) are statistical methods used to identify homogenous groups of patients using a set of descriptive variables. They differ from classification methods (e.g. logistic regression) in that they do not aim to identify pre-specified groups. In case were the number of variables is high, they may be preceded by multivariable descriptive method (e.g. Multiple Correspondance Analysis), which calculate axes, or components, which summarize groups of correlated variables. This allow a more straightforward description of patients, and this lowers the noise in data.

## Results

**Figure 1:**
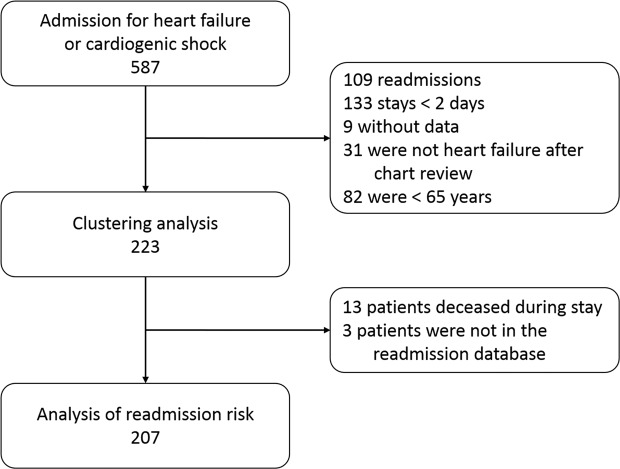
Flow chart of inclusion of Heart Failure cases in analyses (modified from Thesis^21^).

Among the 587 hospitalizations for heart failure in Lariboisière during 2013, 223 patients were included in our clustering analysis (Fig. 1, modified from Thesis^21^). The mean age was 82 +/− 9 years, 54% were women, and 70% lived in Lariboisiere’s catchment area. The patients were mainly admitted from the ER (62%), and the median LOS was of 9 days (quartiles [5–13]). All variables are described in Table 1.

**Table 1:** Description of sample and pathways.

Variable^*^	Total N = 223	Group 1 N = 57	Group 2 N = 15	Group 3 N = 70	Group 4 N = 18	Group 5 N = 63	p-value
Age (years)	**82** +**/− 9**	80 (+**/−** 9)	80 (+**/−** 8)	82 (+**/−** 9)	87 (+**/−** 8)	84 (+/−9)	0.008
Sex (female)	**121 (54%)**	27 (47%)	7 (47%)	33 (47%)	11 (61%)	43 (68%)	0.09
BMI (kg/m², N = 85)	**28** +**/− 6**	27 (+**/−** 5)	24 (+**/−** 4)	28 (+**/−** 5)	25 (+**/−** 1)	33 (+**/−** 9)	0.01
Smoking status (N = 191)	**25 (13%)**	9 (16%)	2 (13%)	3 (4%)	0 (0%)	11 (17%)	—
HBP	**187 (84%)**	49 (86%)	13 (87%)	59 (84%)	13 (72%)	53 (84%)	0.72
COPD	**29 (13%)**	5 (9%)	3 (20%)	4 (6%)	3 (17%)	14 (22%)	0.04
Diabetes	**87 (39%)**	26 (46%)	6 (40%)	30 (43%)	3 (17%)	22 (35%)	0.22
Renal failure	**64 (29%)**	10 (18%)	7 (47%)	16 (23%)	9 (50%)	22 (35%)	0.02
Atrial fibrillation	**124 (56%)**	28 (49%)	5 (33%)	42 (60%)	7 (39%)	42 (67%)	0.05
Hospital near recruitment area	**155 (70%)**	37 (65%)	8 (53%)	43 (61%)	15 (83%)	52 (83%)	0.02
**Syndrome**							
*Acute pulmonary edema*	**107 (48%)**	40 (70%)	10 (67%)	26 (37%)	9 (50%)	22 (35%)	0.0003^†^
*Congestive heart failure*	**106 (48%)**	17 (30%)	5 (33%)	41 (59%)	7 (39%)	36 (57%)	0.005^†^
*Cardiogenic shock*	**4 (2%)**	0 (0%)	0 (0%)	1 (1%)	2 (11%)	1 (2%)	—
*Right heart failure*	**6 (3%)**	0 (0%)	0 (0%)	2 (3%)	0 (0%)	4 (6%)	—
LVEF							0.0005
*Reduced (*≤*40%)*	**67 (30%)**	24 (42%)	5 (33%)	23 (33%)	8 (44%)	7 (11%)	
*Unknown*	**53 (24%)**	5 (9%)	4 (27%)	13 (19%)	5 (28%)	26 (41%)	
BNP value (N = 181)	**851 (380–1489)**	845,5 (395–1424)	2300 (395–3750)	872 (359–1480)	1106 (603–1709)	769 (345–1512)	0.04
Echocardiography	**159 (71%)**	53 (93%)	12 (80%)	57 (81%)	13 (72%)	24 (38%)	<0.0001
Chest X-ray	**133 (60%)**	19 (33%)	6 (40%)	48 (69%)	13 (72%)	47 (75%)	<0.0001
BNP dosage	**181 (81%)**	52 (91%)	3 (20%)	61 (87%)	15 (83%)	50 (79%)	<0.0001
Creatinine dosage	**212 (95%)**	57 (100%)	5 (33%)	70 (100%)	18 (100%)	62 (98%)	<0.0001
Loop diuretics	**161 (72%)**	48 (84%)	12 (80%)	53 (76%)	6 (33%)	42 (67%)	0.0006
ACE-I/ARB	**124 (56%)**	42 (74%)	8 (53%)	44 (63%)	2 (11%)	28 (44%)	<0.0001
BB	**118 (53%)**	40 (70%)	4 (27%)	42 (60%)	0 (0%)	32 (51%)	<0.0001
ACE-I/ARB or BB	**164 (74%)**	52 (91%)	10 (67%)	56 (80%)	2 (11%)	44 (70%)	<0.0001
**Admission mode**							
*Home*	**71 (32%)**	25 (44%)	8 (53%)	35 (50%)	2 (11%)	1 (2%)	<0.0001^†^
*ER*	**138 (62%)**	23 (40%)	6 (40%)	33 (47%)	14 (78%)	62 (98%)	<0.0001^†^
*Other hospital*	**14 (6%)**	9 (16%)	1 (7%)	2 (3%)	2 (11%)	0 (0%)	—
**Discharge mode**							
*Home*	**162 (73%)**	40 (70%)	9 (60%)	60 (86%)	1 (6%)	52 (83%)	<0.0001^†^
*Other hospital*	**21 (9%)**	8 (14%)	3 (20%)	5 (7%)	2 (11%)	3 (5%)	—
*Mid- or long-term care hospital*	**27 (12%)**	8 (14%)	3 (20%)	5 (7%)	3 (17%)	8 (13%)	—
*Death*	**13 (6%)**	1 (2%)	0 (0%)	0 (0%)	12 (67%)	0 (0%)	—
**Number of wards**							
1	**93 (42%)**	9 (16%)	7 (47%)	69 (99%)	5 (28%)	3 (5%)	<0.0001^†^
2	**113 (51%)**	45 (79%)	3 (20%)	1 (1%)	5 (28%)	59 (94%)	—
3	**12 (5%)**	3 (5%)	4 (27%)	0 (0%)	4 (22%)	1 (2%)	—
4	**5 (2%)**	0 (0%)	1 (7%)	0 (0%)	4 (22%)	0 (0%)	—
**Wards**							
*SSU*	**75 (34%)**	7 (12%)	0 (0%)	0 (0%)	8 (44%)	60 (95%)	<0.0001
*Cardiology*	**119 (53%)**	50 (88%)	12 (80%)	44 (63%)	8 (44%)	5 (8%)	<0.0001
*ICCU*	**65 (29%)**	48 (84%)	7 (47%)	1 (1%)	9 (50%)	0 (0%)	<0.0001
*ICU*	**17 (8%)**	2 (4%)	7 (47%)	1 (1%)	6 (33%)	1 (2%)	<0.0001
*Geriatric*	**34 (15%)**	0 (0%)	0 (0%)	4 (6%)	2 (11%)	28 (44%)	<0.0001
Length of stay	**9 (5**–**13)**	7 (5–11)	11 (6–19)	7 (4–11)	16,5 (11–22)	12 (7–16)	<0.0001
Long stay	**63 (28%)**	13 (23%)	6 (40%)	15 (21%)	12 (67%)	17 (27%)	0.002

^*^When variables had missing values, the number of available values were provided.

^†^p-value of a test comparing this modality versus all others. Indeed, for these variables, global tests could not be performed because of low frequencies. Therefore, we only tested specific modalities.

Values are n (col %), mean +/− standard deviation or median (Q1–Q3) depending on the type and distribution of the variable.

Tests are Chi-square, Fisher, ANOVA, or Kruskall-Wallis depending on the type and distribution of the variable.

HBP: high blood pressure. COPD: chronic obstructive pulmonary disease. LEVF: left ventricular ejection fraction. BNP: b-type natriuretic peptide. ACE-I/ARB: angiotensin-converting enzyme inhibitors or angiotensin receptor blockers. BB: beta-blockers. SSU: short stay unit. ICCU: intensive cardiac care unit. ICU: intensive care unit.

The MCA showed that the more discriminant characteristics were the specialization of the in-hospital pathway (cardiology versus other) and the complexity of the in-hospital pathway (measured by the number of wards in the in-hospital pathway).

**Figure 2:**
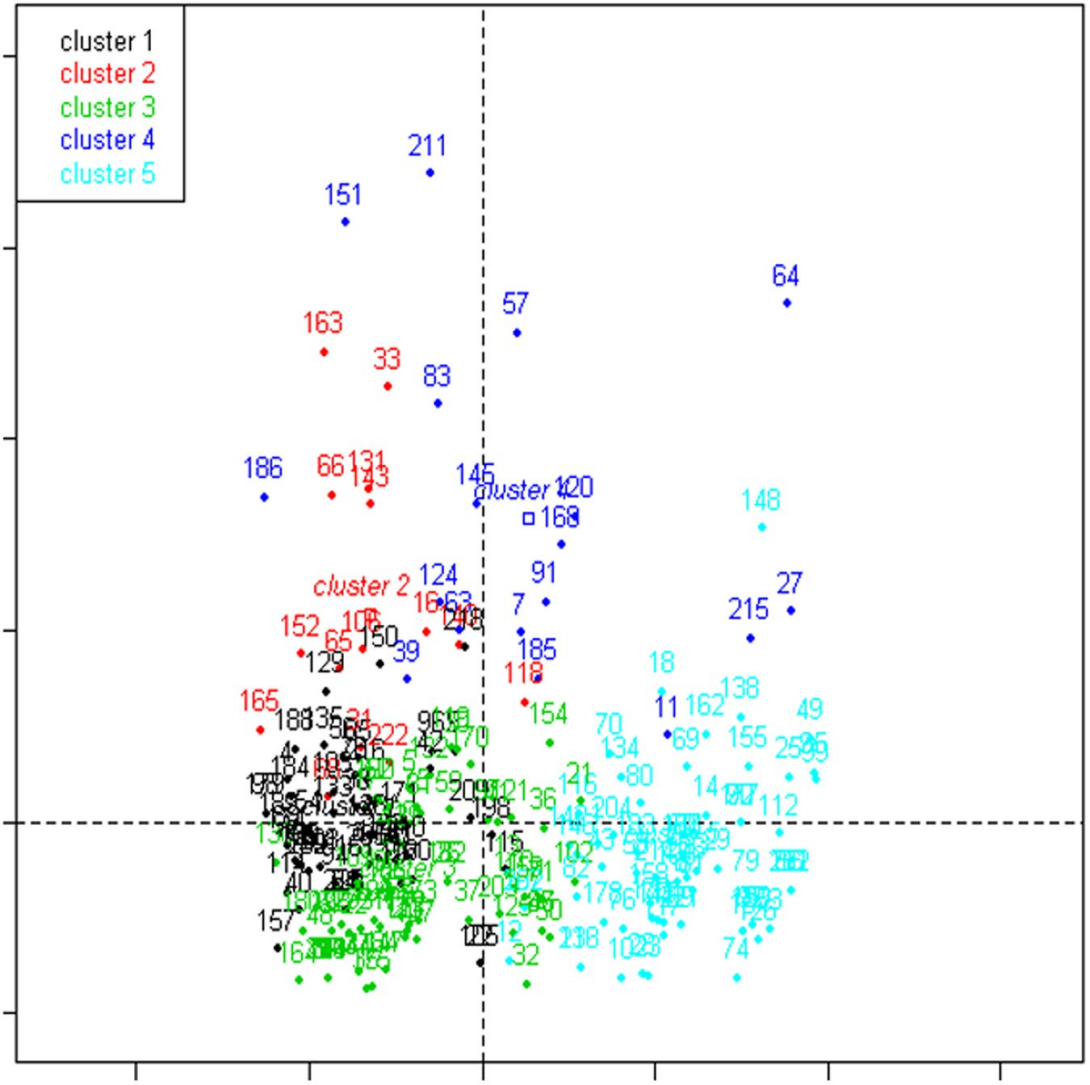
Projection of clusters based on the two first axes of the Multiple Component Analysis (re-used from Thesis^21^). The horizontal axis is the first axis. It opposes pathways with passages in cardiology wards on the left and pathways with passages in geriatrics on the right. The vertical axis is the second axis. It opposes simple pathways on the bottom and complex pathways on the top.

The clustering method provided a 5-group solution. The projection of groups on the two first axes of the MCA is provided in Fig. 2 (re-used from Thesis^21^). Detailed results of the clustering method are provided in the Supplementary data. As we built these groups using clinical characteristics, care management and in-hospital pathway elements, the groups reflect as much the in-hospital care pathways as the disparities in clinical care practices in the different units or etiologies for admission to the different unit. The in-hospital pathway of group 1 (N = 57) typically included a passage through the ICCU followed by the cardiology ward, a BNP and an echocardiograph, and a prescription of at least one pharmacological treatment in 91% of cases. Patients in this group were typically younger, were more often males with fewer comorbidities and presented more often with acute pulmonary edema or a reduced ejection fraction. The in-hospital pathway of group 2 (N = 15), similar to group 1, included stays in cardiology and the ICCU but also in the ICU, leading to a more complex in-hospital pathway and a longer LOS. Surprisingly, the diagnostic management during hospitalization and therapeutic management at discharge was very scarce: only 67% of patients had a prescription of ACE-I, ARB or BB. The clinical characteristics of these patients did not differ from those of group 1, except for a high frequency of renal failure. The in-hospital pathway of group 3 (N = 70) typically included a unique ward, which was often cardiology. Diagnostic and therapeutic management was high, yet not as high as in group 1. Patients in this group had a similar age and comorbidity profile, were often males, and presented often with congestive heart failure. The in-hospital pathway of group 4 (N = 18) was complex, long and not very specialized. Nearly all patients who died during their stay were in this group, which explains why the rate of treatment prescription at discharge was low. The in-hospital pathway of group 5 (N = 63) typically included an admission from the ER to the SSU, followed by a passage through another ward, which was often geriatrics. The diagnostic management was often performed - with the particularity that the chest X-ray was more frequent than an echocardiograph - and an pharmacological treatment was prescribed to 70% of patients (ACE/ARB or BB). Patients in this group were older, more often females with more comorbidities, and presented often with congestive heart failure.

The association between the in-hospital care pathway and readmission rate was assessed for the 207 patients for whom readmission data were available. Overall, at 30 days, 11% and 27% of patients were readmitted at least once for HF and for any cause, respectively. These rates increased to 38% and 75% at one year (Table 2). The in-hospital care pathway had a significant impact on the risk of 1 year readmission for heart failure (p = 0.0015), and this result was primarily due to the data from group 2, which increased this risk (Table 3). The in-hospital care pathway had no significant impact on other risks for readmission (30 day readmission for HF or all cause, 1 year readmission for all cause).

**Table 2:** Number of readmissions (N = 207).

	N	Incidence (95% CI)
30-d readmission for HF	22	11% (6%–15%)
30-d readmission for any cause	55	27% (21%–33%)
1-y readmission for HF	79	38% (32%–45%)
1-y readmission for any cause	156	75% (69%–81%)

**Table 3:** Effect of pathway group on 1-year readmission risk for heart failure.

Group	Parameter estimate	95% Confidence Interval	p-value
1	1 (ref)		
2	1.2955	[0.3829; 2.2082]	0.0054
3	−0.6191	[−1.2829; 0.0446]	0.0675
4	−0.2866	[−1.6906; 1.1174]	0.6891
5	−0.3179	[−1.0267; 0.3909]	0.3793

Binomial negative regression model with offset on the follow-up time, adjusted on COPD, syndrome, and current tobacco use.

## Discussion

In this single-center, retrospective study, we report the building of homogenous groups based on in-hospital care pathways among patients hospitalized for heart failure. Five groups emerged from the clustering method: group 1 was typical of highly specialized cardiology care; group 3 was typical of usual cardiology care; and group 5 was typical of geriatric care with less echocardiography and fewer aetiologic treatments prescribed at discharge. Nevertheless, the latter group had no increased risk of readmission. Group 2 was very similar to group 1 regarding the specialization of the in-hospital pathway and the clinical features of the patients; however, the in-hospital pathways were more complex, and the diagnostic and therapeutic management was scarce. This group had an increased risk of 1-year readmission for heart failure. Group 4 was characterized by complex in-hospital pathways and a low rate of discharge treatments, due to the high rate of deaths in this group.

Although this study is monocentric, our population seems very similar to larger studies. The LOS observed in this study was very similar to a nationwide study on medico-administrative data^22^. Another nationwide study, Ofica^23^, described the spectrum of hospitalized heart failure syndromes and a part of their in-hospital care pathways. We observed comparable ER use and ICU/ICCU use and a similar rate of natriuretic peptide testing. Similar to the findings in the Ofica study, acute pulmonary edema was more often managed in the ICCU or ICU.

Nevertheless, some differences may be noted. We report fewer echocardiographic examinations (71 vs 82%), and the rate of medication prescribed at discharge was slightly lower in our study. Loop diuretics, ACE-I/ARB and BB were prescribed in 72%, 56%, and 53% of patients, respectively, compared to 85%, 68%, and 56% of patients in the Ofica study. We guess that these differences are linked to another notable one: in the Ofica study, 77% of patients were admitted to cardiology or the ICCU, while in our study this in-hospital pathway applied to only 48% of patients, which is certainly due to our exclusion of younger patients. These differences are linked probably because specialized wards tend to have more exhaustive management^24,25^.

All these elements of diagnostic and therapeutic management are usual quality measures in heart failure^26^. The relatively low level that we observe raises the following question: are these lacks in management justified by the absence of formal indications (i.e., preserved LVEF) or more contra-indications in patients hospitalized in noncardiology wards, or, at the contrary are they truly quality failures? Because pay-for-performance systems are growing in several developed countries^27,28^ and rely partly on quality measures, this is a crucial question. Therefore, future studies should properly adjust for case-mix and assess the association between proposed quality measures and clinical outcomes. For example, we wished to describe the prescriptions for patients receiving aetiologic heart failure treatments, because it is a major quality measure of HF-rEF. However, these treatments are not recommended in HF-pEF. Therefore, because the EF data was missing for many patients, we could not assess the pertinence of “missing treatment” as a failed quality measure for these patients. This missing data is a recurrent deviation in the analysis of HF care pathways. This deviation can be overcome when analyzing big data, or, as we did, when assessing case-mix and clinical outcomes. Indeed, we believe that the fact that Group 5 did not have an increased risk of readmission, despite less exhaustive management, shows that these patients are falsely classified by the quality measures. This finding is supported by epidemiological data in which older HF patients more often have a preserved EF. In this case, considering the EF may have corrected the impact of this measuring bias.

In our study, the second group had an increased risk of one-year readmission for heart failure, despite the adjustment for case-mix. The findings from this group are very surprising because the findings do not match any typical HF patients. We do not understand why these patients were prescribed such a scarce diagnostic and therapeutic management. Therefore, we believe that their management was not optimal, which raises several questions. First, was the complexity of the pathway responsible for the deviations in the patients’ diagnostic and therapeutic management? In a simulation study, Saillour-Glénisson *et al*.^29^ showed that adherence to guidelines was higher when a cooperation culture was observed. This cooperation culture could be more difficult to obtain across separate wards, implying that complex pathways are challenging for guideline adherence. Second, were the low prescriptions rate of aetiologic treatment at discharge responsible for an incomplete ambulatory management, leading to an increased risk for decompensation? In a previous study, we showed that after a hospitalization for heart failure, only 31% of patients had follow-up visits with a cardiologist^30^. Given the difficulty of managing this disease for GPs^31^, it is likely that for the remaining patients, deviations in specialized therapeutic management are not corrected after discharge. This hypothesis should be explored in a study assessing both the in-hospital organization and the organization of early follow-up after discharge. Third, could we prevent such deviations or react swiftly when they occur? Coordination between wards could be improved by protocols of care^32^, by developing proximity between physicians from different wards^33^, and by efficient communication tools^34^. However, because uncertainty cannot be totally eliminated in medicine, we should also establish security nets. Checklists are inexpensive, easy to use, and effective^35^, and they could detect patients with incomplete management before their discharge.

Our study has several strengths. First, all charts were manually reviewed by a senior physician. Therefore, the diagnoses were less biased than ICD codes. Indeed, it is known that ICD-10 codes for HF do not perfectly identify HF in medical charts^36,37^. Second, we collected both clinical and organizational data, which is rarely done. Third, we used a heuristic methodology that allowed us to find clinically significant results. Indeed, a classical regression method on our many variables, in this small sample, would not have provided stable results. The preprocessing of variables by the clustering method allowed us to reduce the number of potential predictors, without discarding any of them. Therefore, overfitting was limited. The limits of this study include the single-center and retrospective features. The small size of group 4 and the fact that it included the highest in-hospital death rate prevented us from assessing the impact of this very complex pathway on readmission risks. Therefore, our results need to be replicated.

## Conclusion

The deviations in guideline adherence in the management of hospitalized heart failure patients may have two meanings. In most cases, these deviations concern patients for which guidelines are contra-indicated. However, in some cases, these deviations may indicate real nonoptimal management. Detecting these cases could help providers identify their causes and propose corrective actions.

## Supplementary information

Supplementary information.

## Data Availability

Data are available on request to first author (c-duflos@chu-montpellier.fr).
