# Supplementary material for: Hospitalization for acute heart failure: the in-hospital care pathway predicts one-year readmission
Source: Sci Rep. 2020 Jun 30;10:10644. doi: 10.1038/s41598-020-66788-y (PMC7327074; doi:10.1038/s41598-020-66788-y)
Supplement: Supplementary file 1 — Supplementary information. [file 41598_2020_66788_MOESM1_ESM.docx]

**Supplementary information**

Of

‘Hospitalization for acute heart failure: the in-hospital care pathway predicts one-year readmission’

By

Claire Duflos^1,2^, MD, PhD, Pénélope Troude^3^, MD, PhD, David Strainchamps^1^, MSc, Christophe Ségouin^3^, MD, Damien Logeart^4^, MD, PhD, Grégoire Mercier^1,5^, MD, PhD

^1^ Medico-Economic Research Unit, Medical Information Department, CHU, University of Montpellier, France

^2^ PhyMedExp, U1046, INSERM, Montpellier, France

^3^ Public Health Department, Universitary Hospital Saint-Louis – Lariboisière – Fernand-Widal, AP-HP, Paris, France

^4^ Cardiology Department, Universitary Hospital Saint-Louis – Lariboisière – Fernand-Widal, AP-HP, Paris, France

^5^ CEPEL, University of Montpellier, France

Annex 1 : Explanation of the clustering method.

The Multiple Component Analysis (MCA) is part of multivariate descriptive analysis methods developed in the 1970’s. It allows describing a population for which we have partially correlated variables, without needing to choose a priori between these variables. It converts the set of original variables, which are partially correlated, into a set of variables called principal components, or axes, which are linear combinations of the original variables. The components have two characteristics: first, all components are independent from each other; second, the first component has the highest variance, followed by the second, and so forth, until the whole variance of the population is represented. The relations of axes and original variables are displayed numerically by a correlation matrix and graphically by correlation circles; whenever clinically relevant, one can therefore attribute a clinical meaning to an axis from themeaning of the original variables that strongly correlate with it. The most interesting means to display the percentages of variance of each axis is a scree plot. Pragmatically, by interpreting these axes, one can tell (a) which variables are the most important for describing the population (b) which the original variables are strongly correlated with each other and (c) in which the original variables are mostly independent of each other. These axes also have two advantages, which allow them to be used instead of the original variables to sort the population into clusters. First, because an axis concentrates the variance of the variables that it represents, it has high explanation power. Second, by “summarizing” a group of variables, it is less prone to basal noise and therefore is more stable. Moreover, the last axes, which account for a small amount of variance, can themselves be considered as basal noise. Therefore, as classically performed,we applied our clustering method on the first axes of the MCA. The number of clustering axes was chosen according to the elbow criterion, which recommends keeping all axes which allow to gain a sufficient part of variance. We chose an HAC, because it is a classical clustering method that does not require the determination of a number of clusters a priori. This method starts with clusters defined as the observations themselves. Then, the closest clusters were merged by means of a serial algorithm. Each step of the merging algorithm provided a partition of the population into homogeneous clusters (low within-variability) that were different from the others (high between-variability). These partitions are displayed graphically on a tree diagram, where the height of the branch represents the distance between the clusters; therefore, the user sees at once which partitions have a high discriminative power. If several partitions have a similar discriminative power, the final choice is led by the clinical relevance of these partitions.

Annex 2 : Eigenvalues of the Multiple Components Analysis


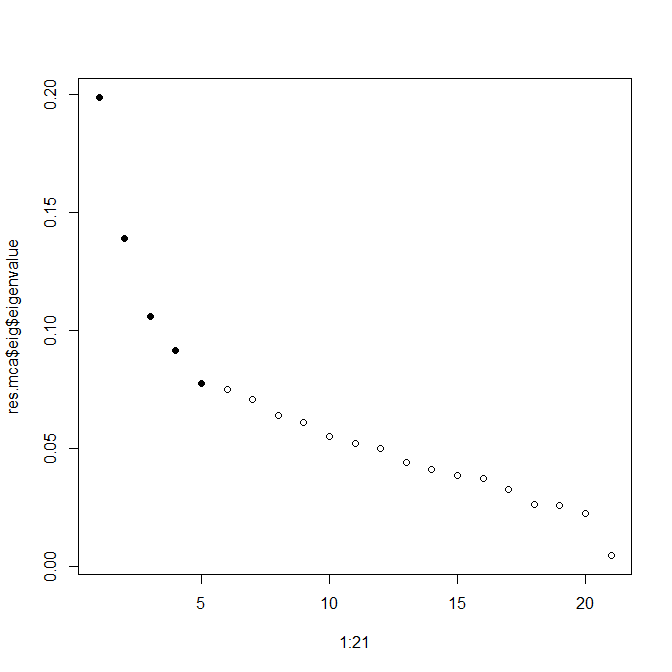


Annex 3 : Results of the Multiple Components Analysis

|  |  | Coordinates | | | | | Contributions | | | | | Cos² | | | | |
| --- | --- | --- | --- | --- | --- | --- | --- | --- | --- | --- | --- | --- | --- | --- | --- | --- |
|  |  | Dim.1 | Dim.2 | Dim.3 | Dim.4 | Dim.5 | Dim.1 | Dim.2 | Dim.3 | Dim.4 | Dim.5 | Dim.1 | Dim.2 | Dim.3 | Dim.4 | Dim.5 |
| Admission mode | Home | -0,725 | -0,109 | 0,666 | 0,141 | 0,225 | 5,264 | 0,171 | 8,347 | 0,432 | 1,294 | 0,246 | 0,006 | 0,207 | 0,009 | 0,024 |
|  | ER | 0,492 | 0,017 | -0,286 | -0,084 | -0,172 | 4,699 | 0,008 | 2,991 | 0,296 | 1,466 | 0,392 | 0 | 0,133 | 0,011 | 0,048 |
|  | Other hospital | -1,167 | 0,391 | -0,559 | 0,11 | 0,551 | 2,685 | 0,431 | 1,158 | 0,052 | 1,535 | 0,091 | 0,01 | 0,021 | 0,001 | 0,02 |
| Discharge mode | Death | 0,211 | 2,028 | 0,598 | -2,156 | 0,335 | 0,082 | 10,79 | 1,233 | 18,51 | 0,527 | 0,003 | 0,255 | 0,022 | 0,288 | 0,007 |
|  | Home | 0,014 | -0,312 | 0,012 | 0,054 | 0,001 | 0,005 | 3,175 | 0,007 | 0,144 | 0 | 0,001 | 0,258 | 0 | 0,008 | 0 |
|  | Other hospital | -0,5 | 0,417 | -0,609 | 0,135 | -0,544 | 0,74 | 0,738 | 2,064 | 0,117 | 2,242 | 0,026 | 0,018 | 0,039 | 0,002 | 0,031 |
|  | Mid- or long-term hospital | 0,202 | 0,569 | 0,111 | 0,61 | 0,257 | 0,155 | 1,764 | 0,088 | 3,08 | 0,642 | 0,006 | 0,045 | 0,002 | 0,051 | 0,009 |
| Number of wards | 1 | -0,431 | -0,31 | 0,814 | -0,139 | -0,421 | 2,434 | 1,804 | 16,337 | 0,55 | 5,939 | 0,133 | 0,069 | 0,474 | 0,014 | 0,127 |
|  | 2 | 0,375 | -0,108 | -0,522 | 0,012 | 0,484 | 2,243 | 0,268 | 8,155 | 0,005 | 9,565 | 0,145 | 0,012 | 0,28 | 0 | 0,241 |
|  | 3 | -0,174 | 2,082 | -1,146 | 1,275 | -1,212 | 0,051 | 10,498 | 4,176 | 5,971 | 6,356 | 0,002 | 0,247 | 0,075 | 0,092 | 0,083 |
|  | 4 | -0,048 | 3,218 | -0,6 | -0,746 | -0,213 | 0,002 | 10,451 | 0,477 | 0,853 | 0,082 | 0 | 0,238 | 0,008 | 0,013 | 0,001 |
| Cardiology | No | 0,801 | -0,055 | 0,191 | -0,046 | 0,128 | 9,393 | 0,063 | 1,007 | 0,067 | 0,615 | 0,56 | 0,003 | 0,032 | 0,002 | 0,014 |
|  | Yes | -0,7 | 0,048 | -0,167 | 0,04 | -0,112 | 8,209 | 0,055 | 0,88 | 0,058 | 0,537 | 0,56 | 0,003 | 0,032 | 0,002 | 0,014 |
| ICU | No | 0,005 | -0,177 | 0,009 | -0,03 | 0 | 0,001 | 1,305 | 0,004 | 0,055 | 0 | 0 | 0,38 | 0,001 | 0,011 | 0 |
|  | Yes | -0,059 | 2,147 | -0,107 | 0,358 | -0,001 | 0,008 | 15,809 | 0,052 | 0,669 | 0 | 0 | 0,38 | 0,001 | 0,011 | 0 |
| SSU | No | -0,579 | 0,022 | 0,256 | -0,048 | 0,002 | 6,988 | 0,014 | 2,578 | 0,106 | 0 | 0,661 | 0,001 | 0,13 | 0,005 | 0 |
|  | Yes | 1,142 | -0,043 | -0,506 | 0,096 | -0,004 | 13,789 | 0,028 | 5,087 | 0,21 | 0 | 0,661 | 0,001 | 0,13 | 0,005 | 0 |
| Geriatry | No | -0,278 | 0,008 | -0,065 | -0,109 | -0,099 | 2,063 | 0,002 | 0,213 | 0,693 | 0,673 | 0,431 | 0 | 0,024 | 0,067 | 0,055 |
|  | Yes | 1,547 | -0,044 | 0,362 | 0,608 | 0,552 | 11,468 | 0,013 | 1,183 | 3,852 | 3,743 | 0,431 | 0 | 0,024 | 0,067 | 0,055 |
| ICCU | No | 0,312 | -0,23 | 0,266 | -0,018 | -0,256 | 2,162 | 1,69 | 2,969 | 0,016 | 3,725 | 0,236 | 0,129 | 0,172 | 0,001 | 0,159 |
|  | Yes | -0,757 | 0,56 | -0,647 | 0,044 | 0,621 | 5,255 | 4,107 | 7,217 | 0,039 | 9,054 | 0,236 | 0,129 | 0,172 | 0,001 | 0,159 |
| BNP dosage | No | 0,356 | 0,488 | 0,678 | 1,239 | 0,011 | 0,749 | 2,02 | 5,124 | 19,736 | 0,002 | 0,029 | 0,055 | 0,107 | 0,356 | 0 |
|  | Yes | -0,083 | -0,113 | -0,157 | -0,287 | -0,003 | 0,174 | 0,469 | 1,189 | 4,58 | 0 | 0,029 | 0,055 | 0,107 | 0,356 | 0 |
| Creatinine dosage | No | -0,687 | 1,314 | 1,27 | 2,664 | -0,086 | 0,731 | 3,831 | 4,704 | 23,907 | 0,029 | 0,024 | 0,09 | 0,084 | 0,368 | 0 |
|  | Yes | 0,036 | -0,068 | -0,066 | -0,138 | 0,004 | 0,038 | 0,199 | 0,244 | 1,24 | 0,002 | 0,024 | 0,09 | 0,084 | 0,368 | 0 |
| Echocardiography | No | 0,992 | -0,081 | -0,009 | 0,176 | -0,095 | 8,872 | 0,084 | 0,002 | 0,61 | 0,209 | 0,396 | 0,003 | 0 | 0,013 | 0,004 |
|  | Yes | -0,399 | 0,033 | 0,004 | -0,071 | 0,038 | 3,571 | 0,034 | 0,001 | 0,245 | 0,084 | 0,396 | 0,003 | 0 | 0,013 | 0,004 |
| Chest X-ray | No | -0,344 | -0,027 | -0,072 | 0,156 | 0,794 | 1,499 | 0,013 | 0,123 | 0,673 | 20,496 | 0,08 | 0 | 0,003 | 0,017 | 0,427 |
|  | Yes | 0,233 | 0,018 | 0,049 | -0,106 | -0,538 | 1,015 | 0,009 | 0,083 | 0,456 | 13,87 | 0,08 | 0 | 0,003 | 0,017 | 0,427 |
| Loop diuretics | No | 0,335 | 0,507 | 0,463 | -0,588 | 0,193 | 0,981 | 3,215 | 3,527 | 6,571 | 0,836 | 0,043 | 0,099 | 0,083 | 0,133 | 0,014 |
|  | Yes | -0,129 | -0,195 | -0,178 | 0,227 | -0,074 | 0,378 | 1,238 | 1,358 | 2,53 | 0,322 | 0,043 | 0,099 | 0,083 | 0,133 | 0,014 |
| ACE-I/ARB | No | 0,376 | 0,431 | 0,395 | -0,156 | 0,19 | 1,969 | 3,706 | 4,104 | 0,739 | 1,285 | 0,113 | 0,148 | 0,125 | 0,019 | 0,029 |
|  | Yes | -0,3 | -0,344 | -0,316 | 0,125 | -0,151 | 1,572 | 2,959 | 3,276 | 0,59 | 1,026 | 0,113 | 0,148 | 0,125 | 0,019 | 0,029 |
| BB | No | 0,164 | 0,436 | 0,352 | -0,19 | 0,155 | 0,4 | 4,025 | 3,456 | 1,163 | 0,907 | 0,024 | 0,169 | 0,111 | 0,032 | 0,021 |
|  | Yes | -0,146 | -0,388 | -0,314 | 0,169 | -0,138 | 0,356 | 3,581 | 3,075 | 1,035 | 0,807 | 0,024 | 0,169 | 0,111 | 0,032 | 0,021 |
| Long stay | No | -0,006 | -0,316 | 0,153 | 0,029 | 0,244 | 0,001 | 3,23 | 0,992 | 0,042 | 3,426 | 0 | 0,254 | 0,059 | 0,002 | 0,151 |
|  | Yes | 0,015 | 0,803 | -0,388 | -0,074 | -0,619 | 0,002 | 8,203 | 2,519 | 0,107 | 8,701 | 0 | 0,254 | 0,059 | 0,002 | 0,151 |

Annex4 : Hierarchical Cluster Analysis performed on the 5 first axes of the MCA


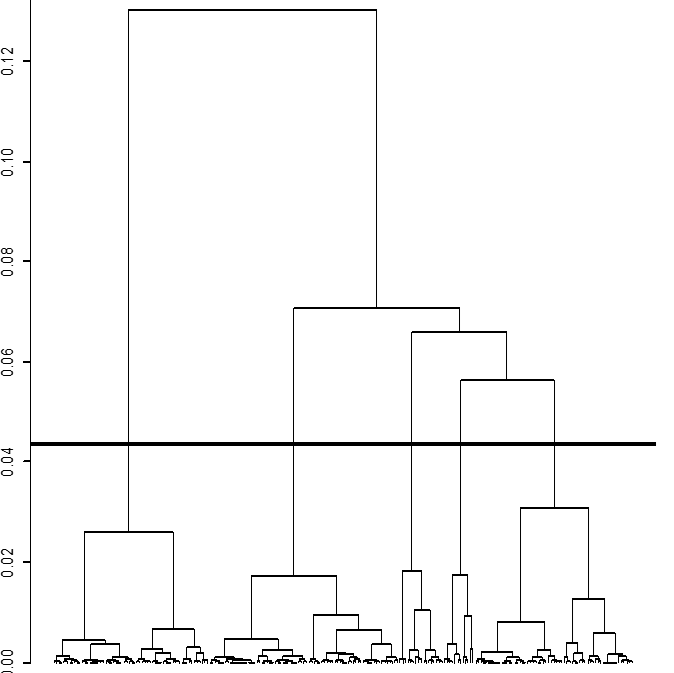


Annex 5 : Variables independently associated with outcomes

|  | Significant variables | p-value |
| --- | --- | --- |
| 30-d readmission for HF | High Blood Pressure | 0.0674 |
|  | Renal failure | 0.0156 |
| 30-d readmission for any cause | Syndrome | 0.0722 |
| 1-y readmission for HF | Groupe | 0.0015 |
|  | COPD | 0.0172 |
|  | Syndrome | 0.0460 |
|  | Smoking status | 0.0428 |
| 1-y readmission for any cause | Diabetes | 0.0459 |
|  | Sex | 0.1003 |

Binomial negative regression model, with offset on the follow-up time, adjusted on significant variables
